# Delays in Newborn Screening for Phenylketonuria from Birth to Diagnosis and Factors Affecting This

**DOI:** 10.3390/children11050571

**Published:** 2024-05-09

**Authors:** Banu Kadıoğlu Yılmaz, Zafer Bağcı

**Affiliations:** 1 Department of Pediatric Metabolism, Konya City Hospital, University of Health Sciences, Konya 42020, Türkiye; 2 Department of Pediatric Nutrition and Metabolism, Faculty of Medicine, Selçuk University, Konya 42250, Türkiye; 3 Department of Pediatrics, Konya City Hospital, University of Health Sciences, Konya 42020, Türkiye; zafer.bagci@sbu.edu.tr

**Keywords:** newborn screening, phenylketonuria, admission time, delay, phenylalanine

## Abstract

This study aims to evaluate the process of neonatal phenylketonuria (PKU) screening from birth to admission to the pediatric metabolism polyclinic, determining delays in the screening program and the factors influencing them. This study was conducted during 2021–2023. Blood collection dates, results, and probable parameters causing delays in the screening program were recorded. This study included 118 infants. Admission time to the polyclinic was (mean ± SD) 25.2 ± 12.6 days (min–max: 3.4–78.9 days). Admission time was significantly high for refugees, those whose parents were consanguineous, and those who had more heel-prick blood samples taken (*p* < 0.001, *p* = 0.005, and *p* < 0.001, respectively). The first heel-prick blood phenylalanine (phe) level was significantly negatively correlated with the admission time (*p* < 0.001). Patients’ admission time whose first blood phe level < 240 μmol/L was statistically significantly higher than in those with ≥240 μmol/L (*p* < 0.001). We determined that there were delays in PKU screening from birth to admission to the polyclinic. Being a refugee, the presence of consanguineous marriages, the increase in the number of heel-prick tests, and blood phe levels at a range of 120–240 μmol/L were the factors that played a role in this delay. Taking steps to reduce the impact of these parameters can prevent delays in newborn PKU screening and increase the success of the screening program.

## 1. Introduction

Newborn screening is a preventive healthcare program that aims to reduce morbidity and mortality by detecting disorders that can be treated with timely intervention before they become symptomatic [1,2]. Within the scope of newborn screening, inherited metabolic diseases; endocrine, hematological, immunological, and cardiovascular diseases; and hearing loss are investigated [1,2]. Newborn screening first began in the 1960s to detect phenylketonuria (PKU) [3]. This process, which started with investigating PKU in newborn screening, has expanded by including new disorders. Today, 34 primary and 26 secondary disorders are screened in the USA with the recommended uniform screening panel (RUSP) [4,5]. In Germany, screening is carried out for 17 target diseases from 6 different pediatric specialties [6,7]. PKU screening in Türkiye started in 1987 and was expanded throughout the country in 1993. PKU and congenital hypothyroidism screening began to be carried out throughout the country on 25 December 2006, within the scope of the national newborn screening program in Türkiye. Today, in addition to PKU, congenital hypothyroidism, biotinidase deficiency, cystic fibrosis, congenital adrenal hyperplasia, and spinal muscular atrophy are other diseases investigated within the scope of newborn screening [8]. PKU is an autosomal recessive inherited metabolic disease caused by a deficiency in the phenylalanine hydroxylase (PAH) enzyme, which converts phenylalanine (phe) to tyrosine (tyr) [9]. PKU disease is caused by biallelic pathogenic mutations in the *PAH* gene [10]. Normal blood phe levels are at a range of 50–110 µmol/L [10]. In the classification based on pre-treatment phe levels, mild hyperphenylalaninemia (HPA) is defined as 120–600 µmol/L, mild PKU is at a range of 600–900 µmol/L, moderate PKU is at a range of 900–1200 µmol/L, and classic PKU is characterized by levels above 1200 µmol/L [11]. Another more straightforward classification of PKU is divided into two categories: forms that do not require treatment and forms that require treatment with BH4, diet, or both [12]. However, classification may not always be that simple. Patients’ phe levels may not have reached their highest level at admission [10]. It is important to analyze plasma amino acids, phe, tyr, and the phe/tyr ratio, as well as the rest of the amino acids to confirm the diagnosis of PKU from positive cases taken from newborn screening [13]. The sensitivity of screening tests increases by including the measurement of the phe/tyr ratio instead of measuring phe alone [14,15]. In cases where phe elevation is detected, the differential diagnosis of tetrahydrobiopterin (BH4) deficiency should be investigated [10]. BH4, the cofactor of the PAH enzyme, has its own biosynthesis and recycling pathway [16]. Defects in this cycle cause six neurometabolic diseases, which are included under the classification of BH4 deficiencies. These diseases are autosomal dominant GTP cyclohydrolase I deficiency (AD-GTPCHD), autosomal recessive GTP cyclohydrolase I deficiency (AR-GTPCHD), 6-pyruvoyl-tetrahydropterin synthase deficiency (PTPSD), sepiapterin reductase deficiency (SRD), Q-dihydropteridine reductase deficiency (DHRPD), and pterin-4-alpha-carbinolamine dehydratase deficiency (PCDD) [16]. Except for AD-GTPCHD and SRD, HPA is the main finding of these diseases [16]. The depletion of monoamine neurotransmitters in the central nervous system and HPA are the main factors in the laboratory and clinical findings of these diseases [16]. While it is important to analyze neopterin, biopterin, and dihydropteridine reductase (DHPR) activity from DBS in the diagnosis of BH4 deficiency, the significant decrease in phe levels at 8–12 h during the BH4 loading test may be an important clue, especially in AR-GTPCHD, PTPSD, and PCDD [16]. The treatment of BH4 deficiencies differs from that of PKU, and early initiation of treatment positively impacts neurodevelopmental outcomes [16]. Therefore, careful differential diagnosis of these diseases is necessary [16]. Apart from BH4 deficiencies, starting early treatment in PKU is also important. If left untreated, phe levels increase in the blood and brain and cause severe neurological and cognitive problems in the patient [12]. The guidelines recommend that phe levels above 360 µmol/L should be treated [12]. While the incidence of PKU is approximately 1 in 10,000 in European countries, it increases to 1 in 4172 and 1 in 4500, respectively, in countries such as Türkiye and Ireland, where PKU is more prevalent [12,13,17]. Current treatments aim to reduce phe levels. There are two main treatment methods: low phe dietary treatment or BH4 treatment [10]. The clinician evaluates and applies these two treatments according to the patient’s BH4 responsiveness and genetic results [11]. The literature generally defines BH4 responsiveness as a 30% decrease in phe level with BH4 loading [11]. Genotype–phenotype correlation has been detected in PKU disease, and it helps predict metabolic phenotypes and BH4 responsiveness from genetic mutations, especially in managing patients with HPA [18,19,20,21]. If two BH4-responsive variants are detected in patients due to genetic analysis, starting BH4 treatment directly without performing a BH4 challenge test is recommended [12]. Early diagnosis and treatment of PKU, which is considered a preventable cause of intellectual disability in the literature, is essential in terms of improving neurological problems and restoring cognitive functions. For this reason, PKU, included in the national newborn screening program by the General Directorate of Public Health in Türkiye since December 2006, has been investigated for every neonate nationwide for 17 years. Within the scope of this screening program, the first heel-prick test is usually taken before discharge from the hospital after birth. We observed in the records of patients referred to the hospital from the newborn screening program that the first blood samples were taken within the first 24 h after birth. However, the literature emphasizes that the optimal time is during 24–72 h after birth since the phe level in the blood increases over time after the newborn is fed rather than immediately after birth [22]. Newborns whose first blood tests are dubious are referred to responsible family healthcare centers and then to pediatric metabolism specialists for verification in the following days, using the algorithm determined by the General Directorate of Public Health [23]. In this group of patients, blood phe level measurement is performed for verification in pediatric metabolism polyclinics. Treatment can be started after the pediatric metabolism polyclinic tests confirm the diagnosis of PKU. The literature recommends that the start of treatment should not exceed ten days after birth [9,12]. However, during patient follow-up, we observed that patients referred from the screening program were admitted to the pediatric metabolism polyclinic later than ten days.

The literature emphasizes the necessity of diagnosing PKU and starting treatment within the first ten days of life [12,23]. Still, when we examined the studies on this subject in the literature, we noticed no study specifically focused on admission times. Again, the literature mentions that there are changes or disruptions in blood collection times in newborn screening due to the increase in early discharges from the hospital [23]. We think the situation should be determined again in today’s conditions, given that many years have passed since the launch of these screening programs and algorithms, which started in the 1960s. Whether revisions are required in the algorithms should be evaluated. Our pioneering study includes newborn patients screened for PKU, referred from both the city and nearby cities. It examines in detail the time taken for these patients to be admitted to the hospital for diagnosis confirmation. Determining the factors affecting the admission time to the pediatric metabolism polyclinic and making improvements in this regard will be beneficial in increasing the success of the screening program.

The aim of this study is to evaluate the factors present in the process of admission to the pediatric metabolism polyclinic for newborns who are at risk for PKU as determined by newborn screening—in particular, the factors present in the process from birth to admission. Thus, we aimed to perform a single-center analysis of the performance of the PKU screening program in Türkiye in its 17th year, according to the timing of blood tests and admission time.

## 2. Materials and Methods

### 2.1. Study Design and Setting

**Figure 1:**
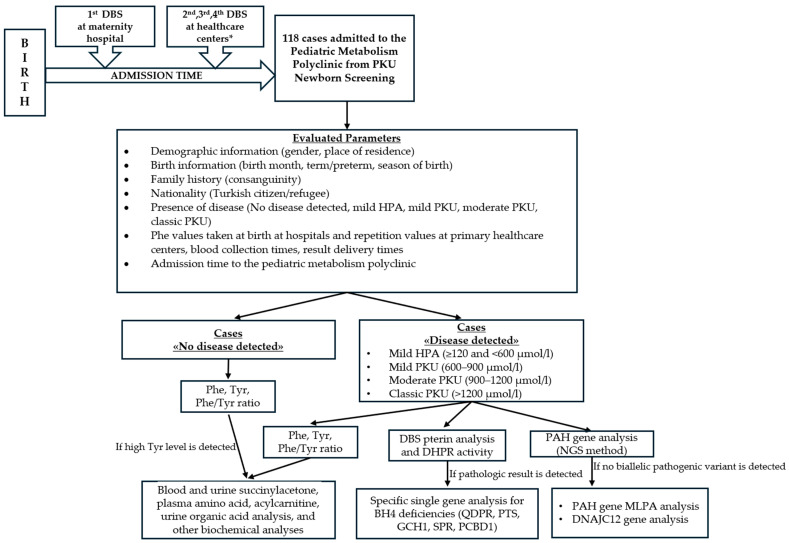
Flow chart of the evaluated parameters of the cases included in this study. *; 2nd, 3rd, and 4th DBS tests are taken according to the algorithm determined by the General Directorate of Public Health of Türkiye, as noted in references [8,23]. PKU, phenylketonuria; HPA, hyperphenylalaninemia; Phe; phenylalanine; Tyr, tyrosine; DBS, dried blood spot; DHPR, dihydropteridine reductase; NGS, Next-generation sequencing; BH4, tetrahydrobiopterin; MLPA, Multiplex Ligation Dependent Probe Amplification.

This study was carried out between 1 April 2021 and 25 April 2023 at Konya City Hospital, which is the only center to which patients and individuals suspected of having PKU are directed within the scope of the national newborn screening program in a city where approximately 2.5 million people live, in Türkiye. This study was conducted on suspected PKU cases from the newborn screening program. It was carried out on newborns in the age group of 0–3 months who were admitted to Konya City Hospital Pediatric Metabolism Polyclinic. This study was conducted as a retrospective, cross-sectional study. The parameters of the factors included in this study that were likely to affect the process from birth to the pediatric metabolism polyclinic were evaluated. The evaluation included demographic characteristics, birth information, family history (consanguinity), nationality, presence of disease, phe levels, and blood test times of those included in this study. The settlements of the study group were evaluated according to the city center where the pediatric metabolism polyclinic is located; they were evaluated from three groups: city center, city districts, and out-of-province. The nationality of the cases was divided into two groups: citizens and refugees. The results of screening for PKU in newborns were evaluated from five groups: those with no disease, mild HPA, mild PKU, moderate PKU, and classic PKU. A definitive diagnosis of PKU was classified according to the literature based on the phe level and other amino acids, including tyr, obtained by analyzing the serum sample taken at admission to the pediatric metabolism polyclinic using the liquid chromatography–tandem mass spectrometry (LC-MS/MS) method [9]. Whether those with the disease had genetic confirmation was also recorded. Phe values of dried blood samples taken from heel-prick tests of the newborns by the principles of the newborn screening program were studied using the LC-MS/MS method. As part of the national newborn screening program, postnatal blood tests were taken within the first 24 h or just before discharge from the hospital. Phe values evaluated in the first heel-prick blood sample test after birth were recorded as the first blood sample phe level. Individuals with a first blood phe level of >120 and <240 µmol/L were referred to primary family healthcare centers to repeat the heel-prick test for blood phe. Patients with phe levels equal to and above 240 µmol/L were directly referred to pediatric metabolism polyclinics. For those with a first blood phe level of >120 and <240 μmol/L, control phe values taken at primary healthcare centers were recorded as the second, third, and fourth blood sample phe levels, respectively. In all groups, blood test collection time, sample result time, and phe values were recorded. The relationship between the study group’s admission time to the pediatric metabolism polyclinic and these parameters, which we think may cause a delay in newborn PKU screening, was analyzed. In our study, we accepted those whose reports obtained from primary screening laboratories included the phrase “unsuitable sample” as “unsuitable samples”. The patients’ phe, tyr, and phe/tyr ratio were evaluated at admission. For patients with phe ≥ 120 µmol/L, Next-Generation Sequencing was used for *PAH* gene variant identification. *PAH* gene deletion duplication analysis (with short tandem repeat fragment analysis and the Multiplex Ligation Dependent Probe Amplification method) and *DNAJC12* gene analysis were also performed on some of the patients with a single heterozygous mutation or no mutation detected in the *PAH* gene. Pterin and DHPR analyses were also performed on dried blood samples in order to assess BH4 deficiency from patients in whom high phe (≥120 µmol/L) was detected. We also performed single gene analysis (*QDPR*, *PTS*, *GCH1*, *SPR*, *PCBD1*) for BH4 deficiency, in addition to differential diagnosis among the patients with pathological results from the pterin and DHPR analyses. For the differential diagnosis of transient neonatal tyrosinemia, tyrosinemia type I, and other tyrosinemias in patients with high tyr (normal range 26.9–275 µmol/L) and high phe, blood and urine succinylacetone, plasma amino acid, acylcarnitine, urine organic acid analysis, and other biochemical analyses were performed. Figure 1 below presents a flow chart summarizing the evaluated parameters of the cases included in this study.

### 2.2. Inclusion and Exclusion Criteria

All male and female infants aged 0–3 months who were born late preterm and near-term at 34 weeks and above with suspected PKU were included in this study. Those with a history of hospitalization after birth (prematurity, sepsis, pneumonia, hyperbilirubinemia, etc.), infants over three months of age, those with chronic diseases (congenital heart disease, chronic liver disease, chronic kidney disease, etc.), patients who had undergone surgery, and patients born under 34 weeks were excluded from this study. Additionally, those with a blood phe level < 120 μmol/L in the blood samples taken at birth and in primary healthcare centers were not included in this study.

### 2.3. Statistical Analysis

Statistical analyses were performed using SPSS 22.0 for Windows. For descriptive criteria, the mean and standard deviation were presented as minimum, maximum, and a percentage of distribution. The conformity of the data for normal distribution was checked with the Kolmogorov–Smirnov test. Student t-tests were used between two groups to compare the means between groups, and One-way ANOVA analysis was performed in more than two groups. Correlation analyses were performed with the Pearson Correlation test. The statistical significance level was taken as *p* < 0.05.

### 2.4. Ethics Statement

This study’s protocols and procedures followed the ethical rules and principles of the Declaration of Helsinki. Before starting this study, ethical approval was obtained from the Necmettin Erbakan University Ethics Committee (Decision No: 2023/4311, date: 5 May 2023).

## 3. Results

### 3.1. Sociodemographic and Clinical Outcomes of Cases

One hundred eighteen cases who met the criteria were included in this study. Sixty (50.8%) of the study group were female, and 13 (11%) were refugees. A total of 53 (45.3%) of the study’s participants lived in the city center. Of the 118 cases in our study, 47 (39.8%) were births in winter. However, when the ratio of PKU cases/total number of births in the month was assessed, the highest ratios were in July (77.8%, 7/9 cases) and June (75%, 3/4 cases), respectively. No disease was detected in 55.1% of the cases. Genetic tests confirmed the diagnosis in 60% of those with the disease detected. There was consanguinity between the parents in 25 cases (21.2%) of the study group. Demographic, birth, and family history information of those included in this study are given in Table 1.

### 3.2. Assessment of Factors Affecting Admission Time

The relationship between the study parameters and the admission time to the pediatric metabolism polyclinic is shown in Table 1. As a result of the statistical analysis, the mean (mean ± SD) time from birth to admission to the pediatric metabolism polyclinic of the 118 patients was 25.2 ± 12.6 days (min-max: 3.4–78.9 days). Admission time to the pediatric metabolism polyclinic for refugees was statistically significantly higher than for those who were citizens (*p* < 0.001). Admission time to the pediatric metabolism polyclinic for those whose parents were related was statistically significantly higher than for those whose parents were unrelated (*p* = 0.005). The admission time to the pediatric metabolism polyclinic was statistically significantly higher in those who had more heel-prick blood samples taken (*p* < 0.001). As we evaluated the admission time of patients whose siblings were being followed up with the diagnosis of PKU, we found that only three of the patients’ siblings were followed up with PKU. The admission times for these three patients were 5 days, 43.5 days, and 14.4 days. The siblings of all three patients were being followed up with mild HPA without treatment.

### 3.3. Assessment of Phe, Tyr, and the Phe/Tyr Ratio of Cases at Admission Time

Table 2 shows the phe, tyr, and phe/tyr ratios of the patients, classified into subgroups as no disease, mild HPA, mild PKU, moderate PKU, and classic PKU, according to the phe level measured at the time of first admission to the pediatric metabolism polyclinic. There were four patients with high blood tyr levels at admission. Three of these were diagnosed as having mild HPA, and one was diagnosed with mild PKU. All tyr levels returned to normal range at follow-up, and all of them were diagnosed with transient neonatal tyrosinemia.

### 3.4. Assessment of Heel-Prick Blood Sampling from Birth to Diagnosis

The first heel-prick blood sample was taken to determine the phe levels of all participants in this study. Of the total, 14 (11.9%) of these tests were unsuitable samples. A second heel-prick blood sample was taken from 102 (86.4%) cases. Of the total, 11 (10.8%) of the second heel-prick blood samples were unsuitable. A third heel-prick blood sample was taken from 76 (64.4%) cases. The fourth heel-prick blood sample was taken from 6 (5.1%) cases. There were no unsuitable samples in the third and fourth blood tests.

The applying day of the heel-prick test in the study group, the phe level, and the resulting time for the samples are listed in Table 3. While the mean sample resulting time (mean ± SD) of the first blood test was 4.62 ± 2.03 days, the mean sample resulting time (mean ± SD) for the fourth blood test was 9.83 ± 1.58 days. We found that as the number of repetitions in blood samples increased, the time it took for the samples to yield results was longer (*p* < 0.001).

### 3.5. Correlation Analysis

In the correlation analysis examining the relationship between the blood phe level and the admission time to the pediatric metabolism polyclinic, a statistically significant negative moderate correlation was detected between the first blood phe level and the admission time to the pediatric metabolism outpatient clinic (*p* < 0.001) (Table 4).

When the correlation between the serum phe level measured in the pediatric metabolism polyclinic and the dried blood sample phe levels measured before admission to the metabolism polyclinic was examined, it was found that there was a moderate statistically significant difference between the phe level measured in the pediatric metabolism polyclinic and the first blood phe level and a highly positive statistically significant difference between the second and third blood phe levels (Table 5).

When the mean admission time to the pediatric metabolism polyclinic for those with the first blood phe level <240 μmol/L and ≥240 μmol/L was compared, it was determined that the mean admission time to the pediatric metabolism polyclinic in those with the first blood phe level < 240 μmol/L was statistically significantly higher than in those with a ≥240 μmol/L (*p* < 0.001) (Table 6).

### 3.6. Further Tests and Follow-Up of Cases

Two patients who were classified as having mild PKU according to their phe level at the time of admission were diagnosed with DHPR deficiency by detecting low DHPR activity, according to the results of DBS pterin analysis and DHPR activity measurement during follow-up. The diagnoses in these patients were confirmed genetically by detecting a homozygous pathogenic mutation in the *QDPR* gene. The nationality of these two patients was Syrian. Except for two patients diagnosed with DHPR deficiency, fourteen patients were receiving BH4 treatment. According to the phe level of these fourteen patients at the time of admission, twelve of them had mild HPA, and two had mild PKU. The phe level of four of these patients was <360 µmol/L at the time of admission, but since it increased above 360 µmol/L during follow-up, BH4 treatment had to be started later. Two of our patients were considered to have classic PKU with a phe level of >1200 µmol/L at the time of admission and were treated with a low phe diet. A patient with a phe level of 174 µmol/L and a phe/tyr ratio of 2.26 at admission was followed up as having mild HPA. Only a heterozygous mutation (c.143T > C [p.Leu48Ser]) was detected in the *PAH* gene of this patient, whose DBS pterin analysis and DHPR activity were normal. During the patient’s follow-up, the phe level was found to be 258 µmol/L at the three-month follow-up but was found to be 1620 µmol/L at the fifth-month follow-up. The patient, who did not respond to the BH4 loading test, was considered to have classic PKU and was followed up with a low-phe diet. No pathogenic variant was detected in this patient’s *PAH* gene MLPA analysis and *DNAJC12* gene.

## 4. Discussion

Newborn screening is a system that includes preanalytical and post-analytical stages rather than a simple test to detect disease [2]. While the preanalytical phase includes collecting the patient’s demographic information, taking blood samples, and sending them to the laboratory, the postanalytical phase consists of conducting the test in laboratories and evaluating and reporting the results [2]. In this process, a timely sample transfer, immediate testing, and short-term results, as well as the availability of confirmatory tests and treatment, play an important role in the success of newborn screening programs [24,25]. In addition to all these factors, ensuring the quality assurance of tests, easy access for patients to healthcare services, strong communication between healthcare providers, and systematic evaluation of patients and test results are other important factors in the success of newborn screening [24,25]. Disruption in these steps will directly affect the success of newborn PKU screening. In our study, we evaluated the performance of the neonatal PKU screening program from the temporal delay window.

In our study, the average (mean ± SD) time to admission to the pediatric metabolism polyclinic of patients who underwent newborn screening for PKU and who needed to be referred to a specialist opinion as a result of the test was found to be 25.2 ± 12.6 days (minimum–maximum: 3.4–78.9 days). This period was far from meeting expectations, with the recommendation that “treatment should be started within the first ten days” [9,12]. Again, in studies conducted in different provinces of Türkiye, PKU patients’ mean age at diagnosis (mean ± SD) was 23.6 ± 19.5 days (minimum–maximum: 2–105 days) and 44 days [26,27]. Although there is no precise study in the literature directly concerning admission times for newborn screening for PKU, in a study conducted in Brazil, more than half of PKU patients were diagnosed within 8–30 days, approximately 30% were diagnosed within the first week, and approximately 5% were diagnosed after a period longer than 30 days [28]. Another study conducted in Jordan, including 294 PKU patients, found that 65.9% of the patients were diagnosed after a period longer than 30 days [29]. Similar to these findings, our study supports the fact that there is a delay in the admission time and diagnosis time of PKU patients in both cities in our country and in different countries.

The timing of blood sample collection may affect the results of newborn screening [30]. In accordance with other studies, it has been reported that the optimal time for taking blood samples should be 24–72 h [22,30]. Our study determined that the first blood tests were taken within 24 h after birth. This may be due to the increasing number of births, the resulting high bed occupancy rates in the hospitals, and patients’ desire to be discharged early. Additionally, in our study, we determined that as the repetition number of blood samples increased, the time it took for the samples to yield results was longer. The reason for this may be that the laboratory where the tests are performed gives priority to the first screening blood tests. Our country has two main centers where blood samples for newborn screening are studied. Screening samples from all over the country are collected in these two centers. The large number of samples to be studied and the small number of laboratories working may cause the first blood tests to be given priority among these samples.

In addition to emphasizing the need for blood samples for newborn screening to be taken between 24 and 72 h of life, some states in the USA recommend a second newborn screening between 7 and 14 days [2]. In our country, a similar practice in newborn screening is applied by taking a second blood sample at primary healthcare centers for those with blood phe levels >120 and <240 μmol/L. In our study, we determined that there was a delay in the admission time for patients with a first blood phe level of >120 and <240 μmol/L compared to those with a first blood level of ≥240 μmol/L. However, in our study, there were cases in which more than two blood samples were taken, although it was not specified in the algorithm. This may be because of the low number of physicians specializing in hereditary metabolic diseases and their absence in certain city centers. The concern that diagnosed patients are often referred to a region far from these settlements may force primary care physicians to repeat blood analysis in patients with blood phe levels >120 and <240 μmol/L. Another reason may be the lack of knowledge of primary care physicians. Parents’ reluctance to go to a distant center may also force primary care physicians to repeat samples. In addition, we found that the unsuitable samples we observed for our study’s first blood samples did not occur in the third and fourth blood samples. When unsuitable first blood samples are considered, it will cause re-testing and prolong the time for dubious cases to be referred to pediatric metabolism polyclinics. This is a factor that reduces the success rate of newborn screening. Unsuitabile samples may result from nurses being unable to properly collect blood in hospitals due to high numbers of patients, lack of information, or problems during transportation. In the future, delays in the admission time can be prevented by increasing the number of pediatric metabolism centers, referring patients with intermediate phe values (>120 and <240 µmol/L) directly to pediatric metabolism polyclinics without unnecessary sample repetitions, and preventing time loss at lower levels in the algorithm. At first glance, it may seem less important that cases with DBS phe levels between 120 and 240 µmol/L be admitted to the pediatric metabolism clinic earlier. However, we know that the diseases in the BH4 deficiency group, which progress to severe neurological findings, most frequently occur in the mild HPA and mild PKU groups [31,32]. Therefore, patients in this group should be admitted to pediatric metabolism polyclinics early to ensure early diagnosis and treatment. The literature states that the prevalence of BH4 deficiencies is 1–2% among HPA cases [31]. In the case series, which includes 626 patients, the most common subgroup of this group is PTPS deficiency, followed by DHPR deficiency [31]. In our study, we found BH4 deficiency in 2 (3.7%) of our 53 patients with high phe. Both of our patients had a DHPR deficiency, which is the second most common disease in the literature and the first in our country [31].

With the introduction of measurement with tandem mass spectrometry (MS/MS) into the routine in newborn screening programs, it has become possible to measure the tyr level to calculate both phe and the phe/tyr ratio [13]. Although blood phe level measurement with the MS/MS technique is much more accurate than older techniques, the phe level detected by this method alone does not always lead to a diagnosis of PKU [13]. An international database includes 133 laboratories where blood for newborn screening is studied; the phe level cut-off is defined as >130 µmol/L, and the phe/tyr ratio is >3 [33]. In the USA, the commonly used cut-off phe level is >120−130 µmol/L, with a phe/tyr ratio of >2 [34]. In our study, we used the cut-off value for phe as >120 µmol/L, but we noticed that some patients with a phe/tyr ratio below 2 were diagnosed with PKU both biochemically and genetically. In the literature, there is information about patients diagnosed with PKU whose phe/tyr ratio is low [35]. In newborn screening, the phe/tyr ratio is important in increasing screening sensitivity, especially when the blood samples are taken within the first 24 h [14,15]. In addition, current studies have shown that it may be useful to predict the need for a phe-restricted diet in the future in infants under BH4 monotherapy alone [36]. The genotype–phenotype relationship in PKU is widely covered in the literature [11,21]. Analysis of patients’ variants for BH4 responsiveness is also very useful in clinical follow-up and for predicting prognosis [11]. However, there are still some unknown points on this subject. For example, there are unexplained situations, such as 81% of the homozygous L48S mutation being BH4-responsive whereas 19% are unresponsive, or some patients with the BH4-responsive E390G homozygous mutation not responding to BH4 [11,37]. In our study, one of the two siblings with the same compound heterozygous mutation received BH4 treatment from birth, and the other was still being followed up with phe level < 360 µmol/L at the age of 10 without BH4 or diet. Although both the phe/tyr ratio and *PAH* gene variant analyses are used to predict prognosis, the fact that there may be exceptional cases should not be forgotten, and the clinical conditions of the patients during follow-up will reveal the actual need for treatment.

Previous studies have determined that the rate of consanguineous marriage in Türkiye is 23.2–24.1% and even rises to 42.6% in regions with low socioeconomic levels [27,38]. In our study, consanguineous marriage was 21.2%. We found a statistically significant delay in admission time in consanguineous marriages. This may be a result of consanguineous marriages being a practice from a low socioeconomic population, insufficient knowledge about PKU disease, and parents’ reluctance to undertake the diagnosis and treatment process. In the future, disseminating visual and public advertisements informing the public about PKU through mass media and organizing training and seminars to educate society about the disease can raise awareness about PKU in this population.

In a study investigating the necessity of newborn screening in refugees, it was determined that two-thirds of refugees were women and children, and most children were born in refugee camps [39]. In the same study, it was emphasized that refugee newborns did not have access to basic health screenings, and diseases such as hypothyroidism and PKU were diagnosed at a late stage [39]. In our study, we found delays in the admission time of refugees to pediatric metabolism polyclinics. In addition to the reasons above, refugees’ socioeconomic losses, language problems, and problems in registration or integration into the health system may also cause a delay in PKU screening. In the future, including refugees in countries’ registration systems and establishing national and international medical technical and logistics collaborations may contribute to timely newborn screening. By the scope of the “Convention on the Rights of the Child” signed by the United Nations in 1989, which states that “children have the right to enjoy the highest attainable standard of health and access to health care services”, we must do our best for the health of all the children in our countries.

There were some limitations in our study. The first limitation was that this study was a retrospective cross-sectional, single-center study involving a local population. Another limitation was that parental literacy and the socioeconomic levels of families were not known. Due to problems with the integration of refugee newborns into the health system and the data recording system, some of the cases with suspected PKU could not be included in this study due to data deficiency.

## 5. Conclusions

In this study, we found that there are still delays from birth to diagnosis in the 17th year of the program screening for PKU in newborns in Türkiye. We determined that being a refugee, consanguineous marriage, increased number of heel pricks, and blood phe levels between 120–240 μmol/L were factors that played a role in this delay. Although the number of mild HPA and mild PKU patients in our study was high, early admission is as important as classic PKU, as these groups also may require treatment and may be diagnosed with BH4 deficiency. Delays in intermediate steps can be prevented by developing training programs on PKU, developing practices to ensure national and international health integration in refugees, and increasing the number of pediatric metabolism centers and specialists. Multicenter and larger population studies are needed on this subject.

## Figures and Tables

**Table 1:** Clinical and sociodemographic characteristics of the cases and the relationship between factors that may affect the admission time.

		Number (*n*)	Percentage (%)	Admission Time (Day)		
				Mean	Standard Deviation	*p* Value
Nationality	Refugee *	13	11	38.16	16.2	<0.001 ^a^
	Citizen	105	89	23.61	11.23	
Gender	Female	60	50.8	23.09	13.15	0.063 ^a^
	Male	58	49.2	27.42	11.82	
Consanguinity	No	93	78.8	23.53	10.93	0.005 ^a^
	Yes	25	21.2	31.51	16.43	
Place of residence	City center	53	45.3	27.51	14.11	0.219 ^b^
	City districts	45	38.5	23.51	10.39	
	Out-of-province	19	16.2	23.16	13.1	
Maturity	Term	95	80.5	25.76	12.54	0.349 ^a^
	Preterm	23	19.5	22.99	13.15	
Birth Month	January	12	10.2	23.55	10.32	0.929 ^b^
	February	17	14.4	23.63	10.69	
	March	8	6.8	28.22	17.34	
	April	9	7.6	31.31	11.81	
	May	11	9.3	25.62	13.37	
	June	4	3.4	25.09	10.6	
	July	9	7.6	25.26	8.87	
	August	4	3.4	23.14	12.34	
	September	6	5.1	21.55	11.18	
	October	10	8.5	21.02	7.91	
	November	10	8.5	27.92	13.83	
	December	18	15.3	25.72	18.06	
Birth season	Spring	28	23.7	28.19	13.84	0.558 ^b^
	Summer	17	14.4	24.72	9.47	
	Autumn	26	22	23.8	11.29	
	Winter	47	39.8	24.41	13.67	
Admission month	January			24.12	14.05	0.776 ^b^
	February			29.62	19.66	
	March			23.64	8.28	
	April			25.2	15.62	
	May			30.19	16.39	
	June			27.94	10.37	
	July			25.63	14.07	
	August			26.93	8.48	
	September			27.74	19.1	
	October			17.94	5.18	
	November			22.65	11.53	
	December			23.72	9.86	
Number of heel-prick blood sample collection	1			14.13	13.74	<0.001 ^b^
	2			20.73	14.19	
	3			28.53	9.65	
	4			36.94	6.73	
The results of screening for PKU in newborns **	No disease	65	55.1			
	Mild HPA	47	39.8			
	Mild PKU	4	3.4			
	Moderate PKU	0	0			
	Classic PKU	2	1.7			
Genetic confirmation	N/A	24	40			
	Assessed	36	60			
	No mutation	3	8.6			
	Heterozygous mutation	7	20			
	Compound Heterozygous mutation	18	51.4			
	Homozygous mutation	7	20			

^a^ Student’s *t*-test; ^b^ One Way ANOVA; PKU, phenylketonuria; HPA, hyperphenylalaninemia; N/A, not assessed; * Refugee: According to the nationality information of the refugees, 11 were Syrian, and 2 were Afghan. **; The definitive diagnosis of phenylketonuria is classified according to the ninth reference, based on the phenylalanine level obtained by studying the serum sample taken at the admission of the pediatric metabolism polyclinic using the LC-MS/MS method.

**Table 2:** Assessment of phe, tyr, and phe/tyr ratio of cases at the first admission to the pediatric metabolism polyclinic.

	No Disease *n* = 65	Mild HPA *n* = 47	Mild PKU *n* = 4	Classic PKU *n* = 2
Phe level (µmol/L)				
Minimum	40.8	124.8	649.8	1440
Maximum	118.8	528	898	2901.6
Mean	80.6	234.4	780	2170.8
Standard Deviation	20.6	105.3	134.5	1033.5
Tyr level (µmol/L)				
Minimum	58.8	42.6	86	61
Maximum	255	741	425	123
Mean	125.1	150.6	216.8	92
Standard Deviation	40.8	106.4	145.6	43.8
Phe/Tyr ratio				
Minimum	0.3	0.2	1.6	23.5
Maximum	2	7.3	10.4	23.7
Mean	0.7	1.9	5.3	23.6
Standard Deviation	0.3	1.1	3.7	0.2

**Table 3:** Assessment of heel-prick blood sample collection days from birth, phenylalanine levels, and delivery time of the results.

	Mean	Standard Deviation	Median	Minimum	Maximum
1st heel-prick blood sample day of collection, day (h)	0.88 (21.1 h)	0.71 (17.04 h)	0.76 (18.2 h)	0	5
1st blood sample phe level (µmol/L)	146.4	83.4	138	18	390
1st blood sample result delivery time (day)	4.62	2.03	4.35	1.25	11.67
2nd heel-prick blood sample day of collection (day)	7.39	4.47	6	2.52	28.87
2nd blood sample phe level (µmol/L)	217.8	177.6	156	120.6	1188
2nd blood sample result delivery time (day)	6.15	2	6.16	2.94	13.38
3rd heel-prick blood sample day of collection (day)	16.98	6.66	15	6.55	39.87
3rd blood sample phe level (µmol/L)	144	42	138	42	318
3rd blood sample result delivery time (day)	7.42	2.97	7.17	2.4	20.17
4th heel-prick blood sample day of collection (day)	23.4	5.26	22.81	16.79	31.88
4th blood sample phe level (µmol/L)	130.8	4.8	132	126	138
4th blood sample result delivery time (day)	9.83	1.58	9.74	8.19	12.11

**Table 4:** Relationship between first heel-prick blood phenylalanine level and admission time.

		Admission Time (Days)
1st blood sample phe level	Pearson Correlation	−0.431 *
	Sig. (2-tailed)	<0.001

* Pearson Correlation test.

**Table 5:** Correlation between the phenylalanine levels at admission time and the heel-prick sample phenylalanine levels.

		Phe Level at Admission Time
1st blood sample phe level	Pearson Correlation	0.558 **
	Sig. (2-tailed)	<0.001
	N	104
2nd blood sample phe level	Pearson Correlation	0.641 **
	Sig. (2-tailed)	<0.001
	N	91
3rd blood sample phe level	Pearson Correlation	0.742 **
	Sig. (2-tailed)	<0.001
	N	76
4th blood sample phe level	Pearson Correlation	−0.033
	Sig. (2-tailed)	0.951
	N	6

** Pearson Correlation test.

**Table 6:** Comparison of the mean admission time to the pediatric metabolism polyclinic between those with the first blood phe level < 240 µmol/L and those with ≥240 µmol/L.

	Admission Time (Days)		
	Mean	Standard Deviation	*p* Value
Phe < 240 µmol/L	27.3	9.9	<0.001 *
Phe ≥ 240 µmol/L	13.4	13.1	

* Student *t*-test.

## Data Availability

Data are available on request due to privacy. The data presented in this study are available on request from the corresponding author. The data are not publicly available because they contain patient identification information.
